# Biochemical and mutational analyses of a *Trametes* pyranose oxidase and comparison of its mutants in breadmaking

**DOI:** 10.1186/s13568-018-0570-y

**Published:** 2018-03-13

**Authors:** Mengzhu Li, Hong Deng, Rui Ma, Huiying Luo, Bin Yao, Xiaoyun Su

**Affiliations:** 1 Key Laboratory for Feed Biotechnology of the Ministry of Agriculture, Feed Research Institute, Chinese Academy of Agricultural Sciences, No. 12 South Zhongguancun Street, Beijing, 100081 People’s Republic of China; 2 Chongqing Academy of Animal Sciences, Chongqing, 402460 China; 3 Key Laboratory of Pig Industry Sciences, Ministry of Agriculture, Chongqing Key Laboratory of Pig Industry Sciences, Chongqing, 402460 China

**Keywords:** Pyranose oxidase, *Trametes*, Breadmaking, Gluten agglomeration

## Abstract

Pyranose oxidase (POx) is a homotetrameric flavoprotein that catalyzes the oxidation of pyranose-configured sugars at position C-2 to corresponding 2-ketoaldoses. The wide substrate specificity makes POx potential for application in various biotechnological industries. In the present study we reported the gene cloning and heterologous expression of a *POx* from the basidiomycete *Trametes* sp. and functionally expressed the gene in *Escherichia coli* BL21(DE3). Based on sequence alignment, three residues were chosen for site-directed mutagenesis to obtain two single mutants (K312E and E539K) and two double mutants (T166A/E539K and K312E/E539K). In comparison to the wild-type, K312E shifted its optimal pH to 5.5 while the optimal temperature of E539K and K312E/E539K increased by 10 °C. The mutants retained more activities over broader pH ranges and higher temperatures and catalyzed d-glucose at higher efficiency (5800‒12,667 M^−1^ s^−1^ for the mutants versus 5083 M^−1^ s^−1^ for the wild-type). The recombinant POx and its mutants were all useful in gluten agglomeration and enlarging the loaf volume, which depends on the amounts of enzymes added. Interestingly, adding the same amount (0.5 nkat/g of flour) of wild-type and mutant enzymes differed in the change of loaf volumes, pinpointing that the catalytic activity is not the sole determinant in applying POx in breadmaking.

**Electronic supplementary material:**

The online version of this article (10.1186/s13568-018-0570-y) contains supplementary material, which is available to authorized users.

## Introduction

Pyranose oxidase (POx) (EC 1.1.3.10) is a large and homotetrameric protein that contains covalently bound flavin adenine dinucleotide (FAD) as a co-factor (Hallberg et al. 2004). It is widely distributed in wood-decaying basidiomycetes but less frequently found in ascomycetes like *Aspergillus* (Pisanelli et al. 2012a; Takakura and Kuwata 2003; Volc et al. 1985). POx catalyzes the oxidation of aldopyranoses at position C-2 to generate corresponding 2-ketoaldoses and releases H_2_O_2_ (Giffhorn et al. 2000). In some cases, it can catalyze the oxidation of C-3 of a few sugars to form 3-keto and 2,3-diketo sugars (Freimund et al. 1998; Volc et al. 2003). The monomeric constituents of plant cell wall polysaccharides such as glucose, xylose, mannose, and galactose and other naturally occurring monomer sugars like sorbose are all substrates of POx (Freimund et al. 1998). POx genes are commonly found in the genomes of lignocellulose-degrading fungi, which have lignin-degrading peroxidases such as lignin peroxidases and manganese peroxidases (Ander and Marzullo 1997; Daniel et al. 1992; Forney et al. 1982; Highley and Murmanis 1985). The released H_2_O_2_ can activate the lignin peroxidases, manganese peroxidase and versatile peroxidase, which in turn are able to oxidize the lignin components in the plant cell wall.

In addition to its important roles in microbial physiology, the unique properties of pyranose oxidase make it potentially useful in many areas including carbohydrate biotransformation (Haltrich et al. 1998), biosensing (Jürgens et al. 1994), and biosynthesis of rare sugars (Granström et al. 2004). POx can also be used in synthetic biology due to its capability to generate a fungal pyrone-antibiotic cortalcerone from glucose (Baute et al. 1987).

In breadmaking industry, POx performs a role similar to glucose oxidase: by introducing disulfide bonds into gluten proteins, it improves the dough strength, rheology, stability, and bread loaf volume (Decamps et al. 2012a, b). With the increasing evidence that POx could be used to improve bread dough stability, it is noticed that many pyranose oxidases tested in breadmaking are limited to those from *Trametes multicolor* (Decamps et al. 2012a, 2013, 2014). In another aspect, although there have been achievements in engineering POx for better thermostability and catalytic efficiency (Bastian et al. 2005; Heckmann-Pohl et al. 2006; Masuda-Nishimura et al. 1999), it is not known how the mutations would affect the behavior of the pyranose oxidase in breadmaking.

To address these issues, in the present study, we cloned a POx-encoding gene from *Trametes* sp., a basidiomycete white rot filamentous fungus that can efficiently utilize lignocellulose. The gene product was heterologously produced in *Escherichia coli*, purified, and biochemically characterized. The gene was further subjected to site-directed mutagenesis and the mutants were compared for their activity and stability and evaluated for their performance in breadmaking.

## Materials and methods

### Strains, plasmids, and reagents

The *E. coli* strains Trans I-T1 and BL21(DE3) from Transgen (Beijing, China) were used for plasmid amplification and expression, respectively. The white-rot fungus *Trametes* sp. is a strain isolated from Shennongjia Nature Reserve (Hubei province, China) (Yang et al. 2011, 2012). The plasmid pET-28a (+) (Invitrogen, Carlsbad, CA) was used for gene expression in *E. coli*. The LA *Taq* DNA polymerase and restriction endonucleases were purchased from TaKaRa (Otsu, Japan). The T4 DNA ligase was obtained from New England Biolabs (Hitchin, UK). The Pfu DNA polymerase was purchased from Tiangen (Beijing, China). The substrates d-glucose, d-xylose, l-sorbose, d-mannose, and d-galactose were purchased from Sigma-Aldrich (St. Louis, MO). All chemicals were of analytical grade and commercially available.

### Gene cloning

*Trametes* sp. was grown at 28 °C in 100 mL of medium containing 20 g/L of glucose, 5 g/L of yeast extract, 5 g/L of peptone, 1 g/L of MgSO_4_ and 2 mg/L CuSO_4_ with constant agitation at 160 rpm. The mycelia were collected on the 5th day for RNA extraction when the *Trametes* sp. culture produced maximum POx activity. The total RNA was extracted using the TRIzol reagent (Invitrogen, Carlsbad, CA) according to the manufacturer’s instructions. The first strand cDNA was synthesized using the First Strand cDNA Synthesis kit (Fermentas, Ontario, Canada). The cDNA of *TsPox* was amplified using the primers *Ts*POxf and *Ts*POxr (Additional file 1: Table S1) and inserted into the expression vector pET-28a(+) between the *Eco*RI and *Not*I restriction sites to obtain the expression plasmid pET-28a-*TsPox*.

### Sequence analysis

Sequence comparisons with known sequences were conducted with BlastP at NCBI (https://blast.ncbi.nlm.nih.gov/Blast.cgi). A multiple amino acid sequence alignment was carried out using the ClustalW program (http://www.ebi.ac.uk/clustalW). The molecular mass of the mature peptide was calculated using Vector NTI 10.0 software (Invitrogen, Carlsbad, CA). No signal peptide was predicted for POx by using the SignalP server (http://www.cbs.dtu.dk/services/SignalP/).

### Construction of mutants

Two single mutants (E539K and K312E) and two double mutants (T166A/E539K and K312E/E539K) were generated by site-directed mutagenesis using pET-28a-*TsPox* as the template, which was carried out by using the Fast Mutagenesis System (Transgen, Beijing, China) according to the instructions of the manufacturer. Specific primers (Additional file 1: Table S1) were used to introduce residue substitution, which was confirmed by DNA sequencing.

### Expression and purification of the POx wild-type and mutants

To express active POx and its mutants, all recombinant plasmids constructed above were individually transformed into the chemically competent cells of *E. coli*. Positive transformants were cultivated in 1 L-flasks at 37 °C and shaken at 200 rpm. When the OD_600_ values reached 0.8‒1.0, IPTG at a final concentration of 1 mM was added to induce the protein expression. After cultivation at 16 °C for an additional 16 h, the cells were harvested by centrifugation at 12,000*g* for 10 min and then re-suspended in a binding buffer (20 mM Tris–HCl, 500 mM NaCl, 10% glycerol, pH 7.6). The cell wall was disrupted by sonication followed by centrifugation at 10,000*g* for 15 min. The supernatant was separated and loaded into a Nickle-NTA (nitrilotriacetic acid) chelating column (GE Healthcare, Uppsala, Sweden). The bound proteins were eluted from the resin with a linear imidazole gradient (40–500 mM) in the binding buffer. Fractions with POx activity were analyzed by the sodium dodecyl sulfate–polyacrylamide gel electrophoresis (SDS-PAGE; 12% separation gel and 5% spacer gel) and the pure fractions were collected and combined. The protein concentration was determined using a Protein Assay Kit (Bio-Rad, Hercules, CA).

### Biochemical characterization

The POx activity was determined spectrophotometrically at 420 nm and 30 °C by monitoring the formation of H_2_O_2_ for 3 min with a peroxidase-coupled assay using 2,2′-azinobis (3-ethylbenzthiazolinesulfonic acid) (ABTS) as the chromogen. The standard assay mixture (total volume, 1 mL) contained 1 mM of ABTS in 50 mM potassium phosphate buffer (pH 6.5), 2 U of horseradish peroxidase, 100 mM of d-glucose, and properly diluted POx sample. One unit (U) of POx activity was defined as the amount of enzyme to oxidize 2 μmol of ABTS per min under the conditions described above.

The pH optimum for the POx activity was estimated at 30 °C in the 200 mM Tris–HCl (pH 2.0–5.0), McIlvaine buffer (200 mM sodium phosphate, 100 mM sodium citrate, pH 6.0–8.0), and 200 mM glycine–NaOH (pH 9.0–12.0). To determine the optimal temperature for activity, the assays were performed at temperatures from 20 to 70 °C at the pH optimum as determined above. For analysis of pH stability, the enzymes were pre-incubated in the 200 mM Tris–HCl (pH 4.0–5.0), McIlvaine buffer (pH 6.0–8.0), or 200 mM glycine–NaOH (pH 9.0) without substrate at 30°C for 1 h, and residual enzyme activities were measured under the standard condition (pH 6.0, 30 °C and 10 min). The thermal stability of the enzymes was determined by measuring the residual activities after incubation at 60 and 65 °C for various durations. The influence of metal ions and chemical reagents on the *Ts*POx activity was tested at concentrations of 5 and 10 mM, respectively.

### Determination of substrate specificity and kinetic parameters

The substrate specificities of wild-type *Ts*POx and its mutants were determined by measuring the enzyme activities at pH 6.5 and 30 °C in the McIlvaine buffer containing 100 mM of d-glucose, d-xylose, d-mannose, l-sorbose or d-galactose as the substrate. Their kinetic parameters were determined in the McIlvaine buffer containing 1‒50 mM of d-glucose under pH 6.5 and 30 °C for 5 min. The *K*_*m*_ and *k*_cat_ values were estimated by fitting the data to the Michaelis–Menten equation using the software GraphPad Prism 5.01 (GraphPad Softwares, La Jolla, CA). The experiments were carried out three times, and each experiment included triplicates.

### Determination of the wheat gluten content

The wheat gluten was checked for residual starch by iodine solution (0.1 g of iodide and 1 g of potassium iodide in 250 mL water). The wheat gluten content was determined according to the State Standard of the People’s Republic of China GB/T 14608-93 (“Method for determination of wet gluten in flour”). The dough was prepared by mixing 100 g of wheat flour and 50 mL of water, followed by extensive washing with water until no gluten was present. The wet gluten was incubated at 105 °C for 12 h to eliminate the bound water. To investigate the effect of POx on gluten agglomeration, 0.25, 0.5, 0.75, and 1.0 nkat/g flour of wild-type *Ts*POx or its mutants was supplemented. The wet and dry gluten were then recorded.

### Application potentials of TsPOx and its mutants in breadmaking industry

The dough was prepared by mixing 100 g of flour, 3.2 g of yeast, 3.0 g of salt, 12.0 g of sugar, 8.0 g of milk powder, 6.0 g of butter, and 120 g of water. The wild-type *Ts*POx or its mutants was supplemented at the dosage of 0.25, 0.50, 0.75 or 1.00 nkat/g flour. Dough mixing was performed at room temperature for 3 min. Mixed dough was then transferred to a lightly greased beaker and incubated at 37 °C in a fermentation cabinet. The fermentation in the breadmaking process continued for 90 min. After fermentation, the dough was punched, transferred to a greased metal baking plate, and incubated at room temperature for a final proof of 45 min. The proofed dough was subsequently baked at 180 °C for 30 min. The loaf volume and weight were measured after cooling down to room temperature for 60 min.

### Nucleotide sequence accession number

The nucleotide sequence of the *TsPOx* gene was deposited in the GenBank database with the accession numbers of MG344741.

## Results

### Gene cloning and sequence analysis

A putative *POx* gene was identified in the draft genome of *Trametes* sp. Its cDNA was predicted to contain 1866 bp and had a G+C content of 64.1%. The gene was named *TsPOx* and the deduced *Ts*POx protein had a calculated molecular mass of 69.2 kDa and a pI value of 6.45. *Ts*POx shared the highest identity (90%) to the POx from *Trametes hirsute* (GenBank accession number: P59097).

### Expression and purification of recombinant TsPOx

**Fig. 1:**
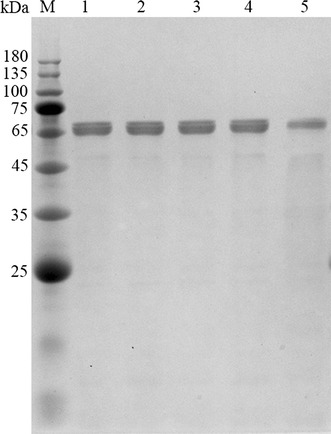
SDS-PAGE analysis of the purified recombinant *Ts*POx and its mutants. M, the molecular mass marker; lane 1, the wide-type; lane 2, the mutant K312E; lane 3, the mutant E539K; lane 4, the mutant T166A/E539K; and lane 5, the mutant K312E/E539K

The cDNA fragment coding for the mature *Ts*POx was ligated into pET-28a(+) to obtain the recombinant plasmid pET-28a-*Tspox*, which was then transformed into *E. coli* competent cells. After IPTG induction at 16 °C for 16 h, the cells were disrupted and the crude enzyme was collected. Recombinant *Ts*POx was purified by immobilized metal affinity chromatography. All recombinant proteins appeared to be composed of two tightly packed bands centering around 69 kDa on the SDS-PAGE gel (Fig. 1), corresponding to the predicted molecular mass. The slightly differing molecular weights of the two bands suggested that the recombinant *Ts*POx could be truncated when it was produced.

### Design, construction and production of mutant enzymes

According to the previous studies on mutagenesis of POx for improved thermostability or broadened substrate specificity (Bastian et al. 2005; Heckmann-Pohl et al. 2006; Masuda-Nishimura et al. 1999), three residues (T166, K312, and E539) of *Ts*POx were selected for mutation (Additional file 1: Figure S1). Two of these mutations have been studied in the *Ts*POx homologs in *Trametes* spp. (Spadiut et al. 2009a, b). The substitutions T166A and K312E correspond to the mutants T158A of P2OxA1 from *Peniophora* sp. (Heckmann-Pohl et al. 2006) and K312E of P2OxB1 from *Peniophora gigantean* (Bastian et al. 2005), respectively, which had increased catalytic efficiencies on glucose, sorbose, and xylose. The substitution E539K is equivalent to the E542K of a POx from *Trametes versicolor* (and *T. multicolor*) with improved thermostability (Masuda-Nishimura et al. 1999; Spadiut et al. 2009a). Therefore, the double mutations T166A/E539K and K312E/E539K were designed in the hope of obtaining mutants with higher catalytic efficiency and better thermostability. By using the recombinant plasmid pET-28a-*Tspox* as template, the four mutant plasmids bearing K312E, E539K, T166A/E539K and K312E/E539K substitutions were obtained. These plasmids were individually transformed into the *E. coli* BL21(DE3) competent cells for heterologous expression and purification. The purified recombinant mutant enzymes showed similar apparent molecular masses to the wild-type *Ts*POx on SDS-PAGE (Fig. 1).

### Effects of pH and temperature on TsPOx activities

**Fig. 2:**
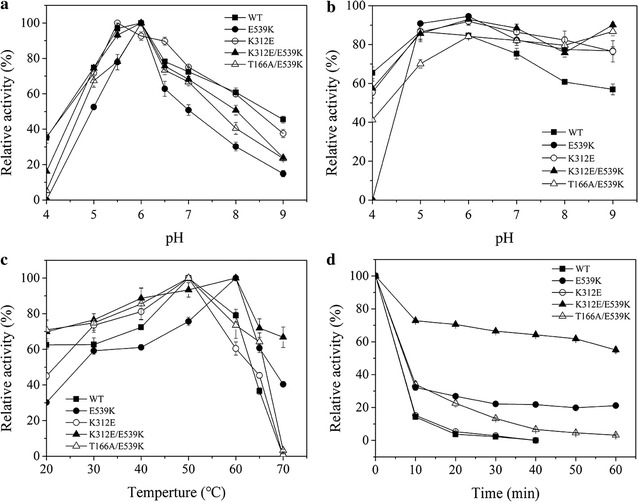
Biochemical characterization of the purified recombinant *Ts*POx and its mutants. **a** pH profile; **b** pH stability; **c** temperature profile; **d** thermostability

The *Ts*POx activities were assayed using d-glucose as the substrate. The purified recombinant *Ts*POx displayed an optimal pH of 6.0 at 30 °C (Fig. 2a), which is similar to other fungal POxes (Table 1). After incubation at pH 4.0–9.0 and 30 °C for 1 h, the enzyme retained more than 56% of its original activity (Fig. 2b). The optimal temperature of *Ts*POx was determined to be 50 °C (Fig. 2c), which falls within the optimal temperature range (40‒60 °C) of most reported POxes (Table 1). The enzyme exhibited good thermostability at 60 °C for 1 h (data not shown) but rapidly lost its initial activity within 40 min at 65°C (Fig. 2d).

**Table 1:** Biochemical properties of *Ts*POx, its mutants, and other pyranose oxidases

Enzyme	Organism	Optimal pH	Optimal temperature (°C)	Specific activity (U/mg)	*k*_cat_ (s^−1^)	*K*_m_ (mM)	*k*_*cat*_/*K*_m_ (M^−1^ s^−1^)	References
*Ts*POx	*Trametes* sp.	6.5	50	6.4 ± 0.8	6.1 ± 0.2	1.2 ± 0.1	5083	This study
E539K		6.0	60	5.3 ± 0.2	7.6 ± 0.5	0.7 ± 0.0	10,857	
K312E		5.5	50	10.2 ± 1.1	8.5 ± 0.0	0.8 ± 0.1	10,625	
K312E/E539K		6.0	60	6.2 ± 0.1	7.6 ± 0.2	0.6 ± 0.0	12,667	
T166A/E539K		6.0	50	2.4 ± 0.0	2.9 ± 0.0	0.5 ± 0.0	5800	
PROD	*Trametes versicolor*	7.0‒7.5	50	12.8	59.9	1.4	42,785	Nishimura et al. (1996)
E542K		N/A	55	13.3	70.6	0.7	100,857	Masuda-Nishimura et al. (1999)
AoPOx	*Aspergillus oryzae*	N/A	N/A	0.58	1.48	2.86	517	Pisanelli et al. (2012a)
AnPOx	*Aspergillus nidulans*	N/A	N/A	1.16	35.44	1.77	20,022	Pisanelli et al. (2012b)
Pyranose oxidase	*Tricholoma matsutake*	7.7‒8.0	50	26.6	111	1.28	86,718	Takakura and Kuwata (2003)
P2Ox	*Peniophora gigantea*	4.5‒6.0	44	0.29	11.9	0.8	14,875	Bastian et al. (2005)
E540K		4.5‒6.0	N/A	13.4	28.5	0.6	47,500	
E540K/312E		N/A	50	N/A	107.6	0.4	269,000	
P2OxA	*Peniophora* sp.	5.0‒6.5	50	2.1	9.4	5.0	1880	Heckmann-Pohl et al. (2006)
E542K		5.0‒6.5	58	4.34	19.4	1.0	19,400	
T158A/E542K		5.5‒6.0	51	29.54	133.48	0.47	284,000	

Among the mutants, K312E had a down-shifted optimal pH to 5.5, while WT and all other mutants had pH optima of 6.0 (Fig. 2a). Besides, the mutants were stable over the pH range of 5.0–9.0 as *Ts*POx, but generally retained greater activities (Fig. 2b). The temperature optima of E539K and K312E/E539K were both 60, 10 °C higher than that of the WT (50 °C) (Fig. 2c). At 65 °C, the mutants E539K, T166A/E539K, and K312E/E539K had 60.7, 64.2, and 71.9% activities respectively, which were higher than that of wild-type *Ts*POx (36.5% of maximal activity) (Fig. 2c). At the temperature of 70 °C, E539K and K312E/E539K showed above 40.3 and 66.7% of maximal activity, respectively, much higher than that of wild-type *Ts*POx (hardly any maximal activity) (Fig. 2c). E539K, T166A/E539K, K312E/E539K showed largely increased thermostability. T166A/E539K, E539K, and particularly K312E/E539K retained more activity than the wild-type when the enzymes were pre-treated at 65 °C for 1 h (Fig. 2d).

### Substrate specificity and kinetic analyses

The wild-type *Ts*POx had the highest specific activity towards d-glucose (6.4 U/mg). When this value was set as 100%, *Ts*POx had moderate activity on d-xylose (55.8%) and l-sorbose (36%), and minor activity on d-mannose (19.7%) and d-galactose (6.5%).

The kinetic parameters of *Ts*POx and its mutants were compared in Table 1. In comparison to WT, the mutants, K312E, E539K, and K312E/E539K had decreased *K*_*m*_ (improved substrate affinity) but increased *k*_*cat*_ (turnover rate) values, which in combination resulted in elevated catalytic efficiencies by 2.1-fold to 2.5-fold. Although the *K*_*m*_ value of mutant T166A/E539K against d-glucose was also decreased, its decreased *k*_*cat*_ (from 6.1 s^‒1^ to 2.9 s^‒1^) accounted for the approximately nearly unchanged catalytic efficiency. The results demonstrated that mutations at positions 166, 312, 539 had effects both on the enzyme properties and catalysis of *Ts*POx.

### Effects of metal ions and chemicals on TsPOx

The influence of a range of metal ions and chemical reagents on the *Ts*POx activity was also tested. Except for SDS and Fe^2+^ that greatly inhibited the *Ts*POx activity, other chemicals had slightly or negligibly negative effects at both concentrations (5 and 10 mM) (Table 2). Some metals (Na^+^, Co^2+^, K^+^, Ca^2+^, Ni^2+^, Mg^2+^, Zn^2+^, and Mn^2+^) even had a more or less stimulatory effect on the activity of *Ts*POx. The great resistance to most metal ions and chemicals may widen the potential application spectrum of *Ts*POx in more industrial fields.

**Table 2:** Effect of metal ions and chemical reagents on the activity of *Ts*POx

Chemicals	Relative activity (%)^a^		Chemicals	Relative activity (%)	
	5 mM	10 mM		5 mM	10 mM
Control	100	100	Cu^2+^	104.9 ± 0.6	101.7 ± 0.8
Ni^2+^	119.5 ± 2.3	125.4 ± 1.7	Zn^2+^	103.5 ± 1.2	123.2 ± 1.6
Mg^2+^	113.0 ± 2.3	120.6 ± 1.6	Cr^3+^	100.0 ± 0.6	61.6 ± 0.8
Mn^2+^	113.0 ± 1.3	123.3 ± 1.1	Ag^+^	94.9 ± 1.2	51.4 ± 1.3
K^+^	110.3 ± 2.2	127.6 ± 1.3	Al^3+^	93.0 ± 0.6	73.0 ± 1.1
Na^+^	108.1 ± 2.3	125.4 ± 1.7	Fe^2+^	11.6 ± 0.3	8.4 ± 0.3
Ca^2+^	106.5 ± 0.6	120.0 ± 0.8	EDTA	85.3 ± 0.7	63.8 ± 2.4
Co^2+^	106.5 ± 2.3	122.2 ± 0.6	SDS	0	0

^a^Values represent mean ± SD (n = 3) relative to the untreated control samples

### Effects of TsPOx on gluten agglomeration in wheat flour

When different dosages of *Ts*POx (0–0.75 nkat/g flour) were added to the wheat flour dough, both the wet and dry weights of gluten more or less increased (Table 3). The best improvement was obtained at the concentration of 0.5 nkat/g flour, with the wet and dry weights of 34.2 and 12.6 g, respectively. However, when increasing the concentration of *Ts*POx to 1.0 nkat/g flour, no elevation in the wet and dry weights of wheat gluten was detected. This suggested that high concentrations of *Ts*POx might have a negative effect on gluten agglomeration. Therefore, the dosage of 0.5 nkat/g flour was used to test the agglomeration effects of *Ts*POx mutants. Of the four mutants, the double mutant K312E/E539K had the greatest performance. The wet and dry gluten weights treated by K312E/E539K were up to 35.5 and 13.2 g, which were significantly higher than those treated by WT *Ts*POx. The results indicated that all POxes tested in this study had effects on gluten agglomeration, and the double mutant K312E/E539K was the best performer.

**Table 3:** Effect of *Ts*POx and its mutants on the wheat flour gluten

	Dosages (nkat/g)	Wet weight (g)	Dry weight (g)
Control	0	32.8 ± 0.3^a^	11.4 ± 0.1^a^
*Ts*POx	0.25	33.4 ± 0.2^d^	12.0 ± 0.2^d^
	0.5	34.2 ± 0.3^b^	12.6 ± 0.1^b^
	0.75	34.1 ± 0.1^b^	12.5 ± 0.1^b^
	1.0	32.0 ± 0.4^c^	10.6 ± 0.3^c^
*Ts*POx	0.5	34.2 ± 0.3^a^	12.6 ± 0.1^a^
E539K	0.5	34.6 ± 0.2^a^	13.0 ± 0.1^b^
K312E	0.5	34.3 ± 0.4^a^	12.9 ± 0.2^a, b^
T166A/E539K	0.5	33.1 ± 0.0^b^	12.6 ± 0.4^a^
K312E/E539K	0.5	35.5 ± 0.4^c^	13.2 ± 0.1^b^

One-way ANOVA (analysis of variance) was used to analyze the values. PROC ANOVA of SPSS 19.0 was applied to identify the differences among groups, and DUNCAN’S test was conducted to examine the differences among the treatments and values. Statistical significance was set at *p *< 0.05. Different letters (^a^, ^b^, ^c^, and ^d^) in the same column mean that there are significant difference between treatments (*p *< 0.05), while the same letters indicate that there are no significant difference between the two values (*p *> 0.05). The number of samples was three for each experiment

### Application of TsPOx in breadmaking

**Fig. 3:**
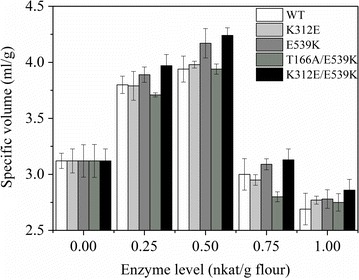
Effect of *Ts*POx wild-type and the four mutants (K312E, E539K, T166A/E539K, and K312E/E539K) and its mutants on the volume of bread loaf. The number of samples was three for each experiment

The wild-type *Ts*POx and its four mutants were tested for their effect on enlarging the loaf volume in breadmaking. Addition of different dosage of *Ts*POx caused change of the bread loaf volume (Fig. 3). For all proteins, adding 0.25 and 0.5 nkat/g flour increasingly enlarged the loaf volume. K312E and T166A/E539K had a similar enlarging effect to that of the wild-type. E539K and K312E/E539K performed better at both dosages. However, when increasing the POx dosages to 0.75 and 1 nkat/g flour, no more improvement was detected in the bread loaf volumes.

## Discussion

POx is widely distributed in lignocellulose-degrading fungi. It catalyzes the oxidation of pyranoses to corresponding keto-sugars and provides hydrogen peroxide to class II peroxidases. With broad substrate specificity and H_2_O_2_-producing capability, POx has much application potential in many areas. However, unlike the other enzymes such as glycoside hydrolases or laccase involved in lignocellulose degradation, much less attention has been paid on POx regarding either its gene diversity or application. In this study, we explored the genome of a *Trametes* sp. and discovered a pyranose oxidase gene with homology to other pyranose oxidases. The conservation of residues important for thermostability and catalytic ability enabled us to fast design, construct, and test the mutants with substantially improved characters in breadmaking.

For the *T. versicolor* POx E542K mutant, an elevated optimal temperature and lower *K*_*m*_ were observed, which is also the case for E539K of the *Ts*POx (Table 1 and Fig. 2). As shown by the results, the mutant K312E had a decreased *K*_m_ and increased *k*_cat_, which resulted in a ~ 2.5-fold improvement in the catalytic efficiency. Combining K312E and E539K further decreased the *K*_*m*_ to 0.6 mM, similar to that observed in the *P. gigantea* P2OxB2H (Bastian et al. 2005). However, this combination did not have much effect on the turnover number. Addition of another mutation T166A did not have a beneficial effect on the enzyme regarding catalytic ability and thermostability. The *K*_*m*_ of the T166A/E539K decreased to 0.6 mM, similar to that observed for the *Peniophora* sp. P2OxA2H (Heckmann-Pohl et al. 2006). It suggested that the additive effects of combinatorial mutations would depend much on the context of the mutated residues even in pyranose oxidase homologs with high amino acid sequence similarity.

Glucose oxidase and POx have a similar role in oxidizing glucose and producing hydrogen peroxide as one of the products, which is widely believed to be at least partially accounting for their roles in breadmaking (Bonet et al. 2006; Decamps et al. 2014). In the present study, low dosages of *Ts*POx and its mutants had significant enlarging effects on the bread loaf volume, but high dosages of enzymes didn’t. Similar results have been observed in glucose oxidase or POx in changing dough rheology (Bonet et al. 2006), extracting glutenin (Decamps et al. 2013), and enlarging the loaf volume in breadmaking (Decamps et al. 2012a). Unexpectedly, we observed that even when equal dosage (0.5 nkat/g of flour) of POx was added in breadmaking, the enlarging effect was quite different. Currently, the reason(s) are not known for the observed differences in breadmaking with equal dosages of POx. However, we noticed that, the best performers E539K and K312E/E539K in breadmaking were also most outstanding in gluten agglomeration. E539K and K312E/E539K were the two mutants with elevated optimal temperature (Fig. 2c) and enhanced thermostability (Fig. 2d). Therefore, these results collectively might suggest that, first, since breadmaking involved a heating step (baking), the improved thermostability would allow E539K and K312E/E539K in catalyzing more efficient crosslinking during this process, thereby enlarging the loaf volume to a higher extent. Second, enhanced thermotolerance is well-known to be positively related to increased resistance to stress conditions (Owusu and Cowan 1989). E539K and K312E/E539K could also be more stable in the dough environment than the wild-type and other mutants, which resulted in their excellent performance. Adding more enzymes may cause unwanted reactions which could mask the positive effect of the POx. These hypothesis, however, need to be explored in detail in the future. Our study for the first time pointed out that the biochemical properties of POx may affect its behavior in gluten agglomeration and breadmaking. It is also suggested that either wild-type pyranose oxidases identified from microbes or those artificially evolved require an evaluation before their real application in the bread industry.

## Additional file

**Additional file 1.** Amino acid sequence alignment of *TsPox* with selected pyranose oxidases and primers used in this study.

## Abbreviations

POx: pyranose oxidase
FAD: flavin adenine dinucleotide
H_2_O_2_: hydrogen peroxide
IPTG: isopropy-β-d-thiogalactoside
ABTS: 2,2′-azinobis (3-ethylbenzthiazolinesulfonic acid)
