# Supplementary material for: Biochemical and mutational analyses of a Trametes pyranose oxidase and comparison of its mutants in breadmaking
Source: AMB Express. 2018 Mar 13;8:38. doi: 10.1186/s13568-018-0570-y (PMC5849585; doi:10.1186/s13568-018-0570-y)
Supplement: Supplementary file 1 — Additional file 1. Amino acid sequence alignment of TsPox with selected pyranose oxidases and primers used in this study. [file 13568_2018_570_MOESM1_ESM.docx]

**Additional file 1**


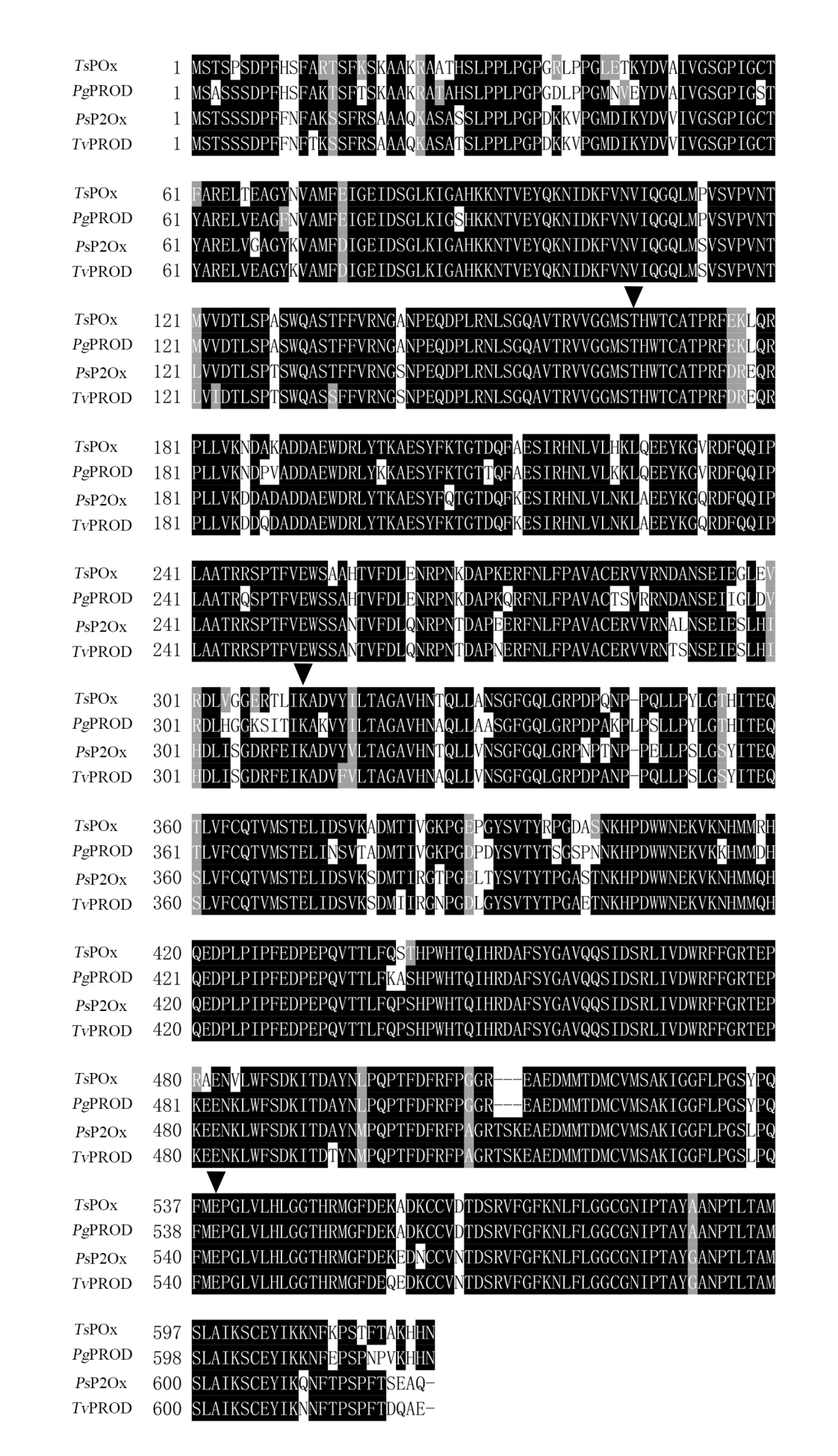


**Figure S1**. Amino acid sequence alignment of *Ts*POx with selected pyranose oxidases *Pg*PROD from *Phlebiopsis gigantean* (GenBank accession number: Q6UG02), P2Ox from *Peniophora* sp. (GenBank accession number: Q8J136), and *Tv*PROD from *Trametes versicolor* (GenBank accession number: D73369). The residues chosen for site-directed mutagenesis are indicated with black triangles.

**Table S1. Primers used in this study**

| **Primers** | **Sequences (**5′→-3′**)^a^** | **Use** |
| --- | --- | --- |
| TsPOxf | GGAATTCATGTCGACCAGCCCGAGCGA | Cloning |
| TsPOxr | AAGGAAAAAAGCGGCCGCTCAGTTGTGGTGCTTCGCGGTG | Cloning |
| E539Kf | TACCCGCAGTTCATGAAGCCTGGTCT | Mutagenesis |
| E539Kr | TCATGAACTGCGGGTAGGAGC | Mutagenesis |
| K312Ef | GAGCGCACGCTCATCGAGGCGGACGT | Mutagenesis |
| K312Er | CGATGAGCGTGCGCTCGCCC | Mutagenesis |
| T166Af | AGCGGTCAGGCGGTCGCCCGCGTCGTC | Mutagenesis |
| T166Ar | CGACCGCCTGACCGCTAAGG | Mutagenesis |

**^a^**The *Eco*RI and *Not*I restriction sites are underlined.
